# Genome-wide functional analysis on the molecular mechanism of specifically biosynthesized fluorescence Eu complex

**DOI:** 10.18632/oncotarget.18914

**Published:** 2017-07-01

**Authors:** Jing Ye, Xiawei Dong, Xuerui Jiang, Hui Jiang, Chen-Zhong Li, Xuemei Wang

**Affiliations:** ^1^ School of Biological Science and Medical Engineering, State Key Laboratory of Bioelectronics, Southeast University, Nanjing 210096, China; ^2^ Nanobioengineering/Bioelectronics Lab, Department of Biomedical Engineering, Florida International University, Miami, FL 33174, USA

**Keywords:** molecular mechanisms, fluorescent Eu complex, GO analysis, KEGG pathway, whole-genome expression profiles

## Abstract

Fluorescence imaging as an attractive diagnostic technique is widely employed for early diagnosis of cancer. Self-biosynthesized fluorescent Eu complex *in situ* in Hela cells have realized specifically and accurately fluorescence imaging for cancer cells. But the molecular mechanism of the *in situ* biosynthesized process is still unclear. In order to reveal this mechanism, we have investigated whole-genome expression profiles with cDNA microarray, incubated with Eu solution in Hela cells for 24 h. Methylthiazoltetrazolium (MTT) assay and laser confocal fluorescence microscopy study showed the low cytotoxicity and specifically fluorescence imaging of Eu complex in Hela cells. It is observed that 563 up-regulated genes and 274 down-regulated genes were differentially expressed. Meanwhile, quantitative RT-PCR was utilized to measure the expression of some important genes, which validated the results of microarray data analysis. Besides, GO analysis showed that a wide range of differential expression functional genes involved in three groups, including cellular component, molecular function and cellular biological process. It was evident that some important biological pathways were apparently affected through KEGG pathway analysis, including focal adhesion pathway and PI3K (phosphatidylinositol 3′ -kinase)-Akt signaling pathway, which can influence glycolytic metabolism and NAD(P)H-oxidases metabolic pathway.

## INTRODUCTION

In modern societies, cancer is one of the most dreaded disease which is very difficult to survive. It is known that various kinds of cancers were found with no clinical symptoms in the early stages, thus most patients died of missing the best timing of treatment [1]. Recently, fluorescence imaging was widely explored for the early detection of the cancer cells, due to its high sensitivity and specificity [2–4]. It has been reported that *in situ* biosynthesized fluorescent agents in cancer cells could be used for tumor targeting imaging [5, 6]. Rare earth elements as unique metal elements have narrow emission line and large separation between excitation-emission [7]. Especially for europium (Eu) species has been utilized for the sensitive detection of cancers [8], because the unfilled f and d molecular orbits can readily result in special optical properties. Considering of those observations, in this contribution we have explored the special biosynthesis strategy of Eu complex for fluorescence bioimaging of cancer cells [9].

However, the relevant mechanisms of the *in situ* biosynthesized process have not been rigorously studied. Hence, in this study we have tried to explore the possible molecular mechanisms of the biosynthesized process and how the fluorescent agents synthesized in cancer cells, which can result in some changes on the genomic level.

At present, many molecular biological methods have been used for exploring the interactions between cell and biomaterial, for instance, Western blotting [10], Real-Time qPCR [11], *in situ* hybridization, ELISA, and others. But they still have some limitations, such as only allowed to analyze several genes at one time. With the development of bioinformatics and high-throughput technologies, microarray technology has been applied in a wide range of biomedical research, which can analyze global genome expression simultaneously [12], and effectively improved comprehending of the molecular mechanisms based on biological processes [13].

So far, microarray technology was utilized in many fields, such as cell-biomaterial interaction in cells or animals, toxicity research, and so on [14–16]. Meanwhile, with the rapid development of the bioinformatics, it has provided more efficient ways to analyze the gene expression data and give accurate interpretation of relevant pathway and gene ontology (GO). GO project provides a controlled vocabulary to describe gene and gene products in a generic cell. The ontology contains three domains: molecular function, biological process, cellular component, which can provide a common descriptive framework. Yet it cannot find more information with the pathway for gene and gene products [17]. Pathway analysis is a functional analysis mapping genes to KEGG pathways, which can further identify minor changes in expression through representing crosstalk networks of some important signaling molecules. Biological pathways play more important role in cell growth and metabolism. The changed biological pathways in cells can well reveal the molecular mechanisms of interactions between cell and biomaterials. Therefore, through these analysis, the researchers could obtain the fully integrated information, including either functional annotations or pathway, in order to effectively and comprehensively characterize the relevant biological processes [18].

In order to explore the special biosynthesis strategy of Eu complex for fluorescence bioimaging of cancer cells, we have further explored the relevant molecular mechanism of the *in situ* biosynthesized process through the analysis of the whole-genome expression profiles with cDNA microarray by using Hela cells incubated with Eu ions. This study would provide a new, convenient, and reliable method to research the mechanisms of the *in situ* biosynthesized fluorescent probes in cancer cells, which could be also benefit to the understanding and further application of the *in vivo* biosynthesis of the relevant fluorescent complexes for target bioimaging of cancer cells/tissues.

## RESULTS

### Cytotoxicity assay

**Figure 1:**
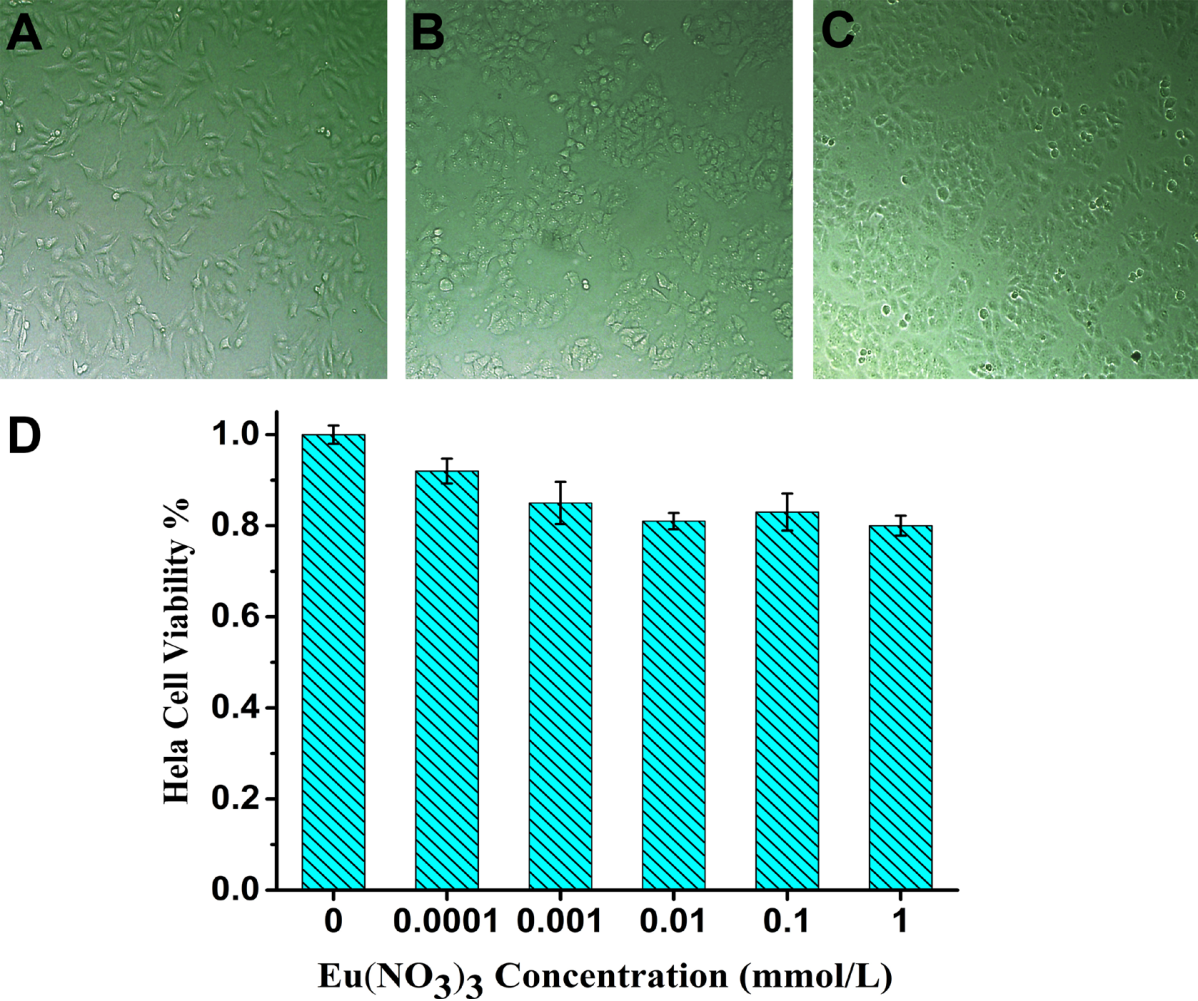
MTT assay assessment after incubation with various concentration of Eu(NO_3_)_3_ solutions for 24 h Cell morphology of Hela cell treated with 1mmol/L (**A**), 0.01 mmol/L (**B**) and 0.0001 mmol/L (**C**) of Eu(NO_3_)_3_ solutions visualized by light microscopy. (**D**) MTT assay assessment of dose-dependent cell viability.

As shown in Figure 1, the cytotoxicity assay of relevant cell treatment with various concentration of Eu(NO_3_)_3_ solution indicates the nice cell growth states after incubation with 1 mmol/L, 0.01 mmol/L and 0.0001 mmol/L Eu(NO_3_)_3_ solution for 24 h, respectively (Figure 1A–1C). It is noted that no significant differences for the Hela cell viability with the presence of 0.0001 to 0.1 mmol/L Eu(NO_3_)_3_ solution (Figure 1D), and the cellular viability was greater than 83%. MTT assay demonstrated the low cytotoxicity of incubated with Eu(NO_3_)_3_ in Hela cells.

### Confocal fluorescence imaging

**Figure 2:**
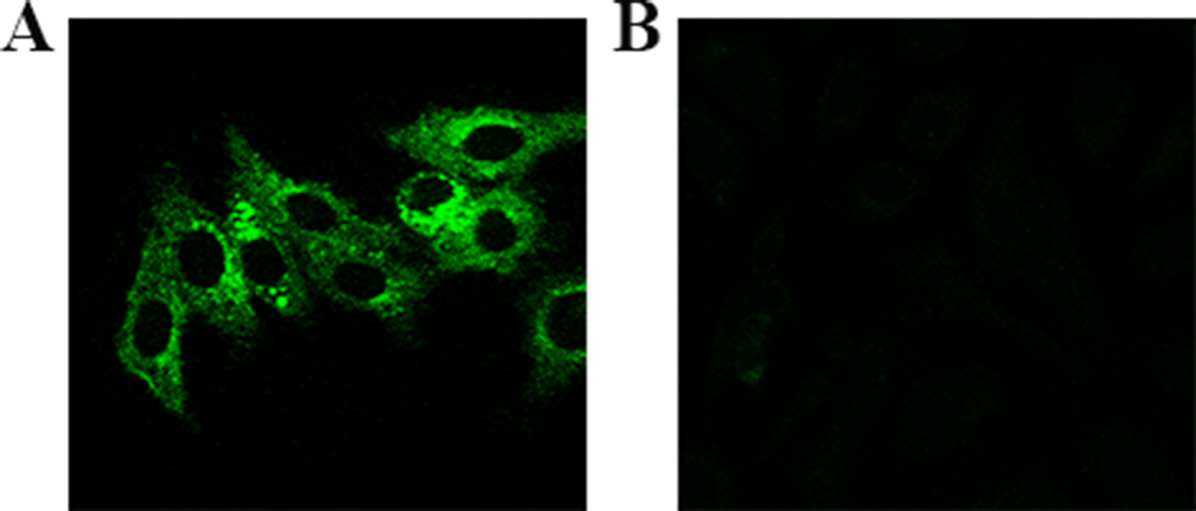
Laser confocal fluorescence imaging of cells after incubated with Eu(NO_3_)_3_ solution (0.01 mmol/L) for 24 h (**A**) Hela cells, (**B**) L02 cells.

The fluorescence bio-imaging of live cells was measured by confocal fluorescence microscopy with excitation wavelength at 488 nm. As shown in Figure 2, the distinct fluorescence signal can be observed in the Hela cells and no obvious fluorescence signal in normal cells (L02 cell), after incubation with the Eu(NO_3_)_3_ solution for 24 h. Fluorescence Eu complex was spontaneously biosynthesized *in situ* with Eu(NO_3_)_3_ solution incubating in Hela cells.

### RNA quantification and gene expression profiling

**Figure 3:**
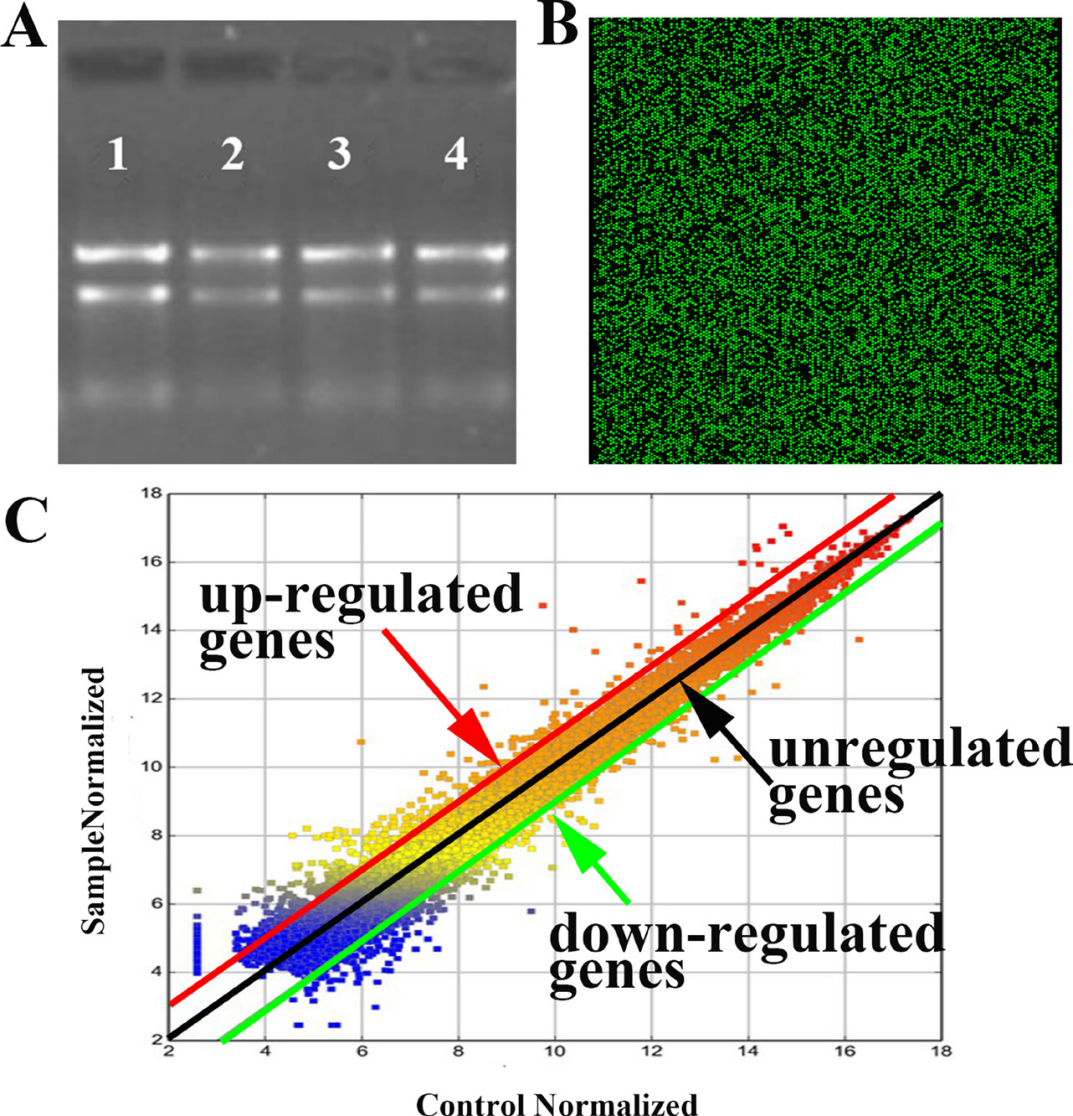
Results of microarray analysis with Eu(NO_3_)_3_ solution (0.01 mmol/L) incubation in Hela for 24 h (**A**) RNA Integrity and gDNA contamination test by Denaturing Agarose Gel Electrophoresis.1: control; 2-4: duplicated samples. (**B**) A partial scanning image of hybridization of microarray with samples; the green bright spot indicates expression of genes. (**C**) Scatter plots of the expression genes with samples vs. control. Genes were classified into three categories: up-regulated (upon the red line), down-regulated (below the green line) and unregulated genes (between red and green line).

To further investigate the mechanism of self-biosynthesis fluorescent Eu complex, we have utilized the analysis of the whole-genome expression profiles with gene microarray. Electrophoresis on a denaturing agarose gel showed the appearances of 5S band, clear 28S and clear 18S rRNA bands (eukaryotic samples), suggested that there is no obvious denaturation of RNA. And the 28S rRNA band was approximately twice as intense as the 18S rRNA band, indicating that the RNA is complete (Figure 3A).

According to filtering criteria, the number of differential expressed genes was 837, which expressions were at least 2-fold changes, including 563 up-regulated genes and 274 down-regulated genes. The up-regulated genes were much more than the down-regulated genes. Figure 3B shows the partial scanning image of hybridization of the microarray, while Figure 3C indicates the scatter plots of the microarray expression data. It is noted that the expression of synovial sarcoma X breakpoint 2 (SSX2) was the highest among the up-regulated genes, incubated with Eu(NO_3_)_3_ in Hela cells for 24 h, compared with the control groups, when Hela cells incubated with DMEM (Table 1). Among down-regulated genes (Table 2), the most significant down-regulated gene is SERPINA3. Meanwhile, the expressions of many other genes were also changed, such as the up-regulation genes of ZNF217 and E2F7.

**Table 1:** The partial of up-regulated genes in Hela cells incubation with Eu(NO_3_)_3_ solution (0.01 mmol/L) for 24 h

GeneSymbol	Fold Change	Genbank Accession	Genomic Coordinates	Description	UniGeneID
**SSX2**	20.1452807	NM_175698	chrX:52787003-52787062	Homo sapiens synovial sarcoma, X breakpoint 2 (SSX2), transcript variant 2, mRNA	Hs.661107
**ZNF217**	10.4316351	NM_006526	chr20:52185714-52185655	Homo sapiens zinc finger protein 217 (ZNF217), mRNA	Hs.155040
**GOLGB1**	10.056337	NM_004487	chr3:121412749-121412690	Homo sapiens golgin B1 (GOLGB1), mRNA	Hs.213389
**E2F7**	9.0852971	NM_203394	chr12:77417635-77417576	Homo sapiens E2F transcription factor 7 (E2F7), mRNA	Hs.416375
**LOC100131067**	8.1123337	NR_034121	chr5:80535122-80535063	Homo sapiens uncharacterized LOC100131067 (LOC100131067), transcript variant 1, non-coding RNA	Hs.655855
**L1CAM**	7.8885547	NM_024003	chrX:153137700-153137641	Homo sapiens L1 cell adhesion molecule (L1CAM), transcript variant 2, mRNA	Hs.522818
**YWHAE**	7.7030992	NM_006761	chr17:1257587-1257528	Homo sapiens tyrosine 3-monooxygenase/tryptophan 5-monooxygenase activation protein, epsilon polypeptide (YWHAE), transcript variant 1, mRNA	Hs.513851
**FBXL8**	7.1064005	NM_018378	chr16:67197815-67197874	Homo sapiens F-box and leucine-rich repeat protein 8 (FBXL8), mRNA	Hs.710714
**CAPZA1**	7.0774027	NM_006135	chr1:113212885-113212945	Homo sapiens capping protein (actin filament) muscle Z-line, alpha 1 (CAPZA1), mRNA	Hs.514934
**LOC649395**	7.0099765	NR_029404	chr7:63894868-63894928	Homo sapiens tyrosine 3-monooxygenase/tryptophan 5-monooxygenase activation protein, epsilon polypeptide pseudogene (LOC649395), non-coding RNA	

**Table 2:** The partial of down-regulated genes in Hela cells incubation with Eu (NO_3_)_3_ solution (0.01 mmol/L) for 24 h

Gene Symbol	Fold Change	Genomic Coordinates	Genbank Accession	Description	UniGeneID
**SERPINA3**	−12.5194476	chr14	NM_001085	Homo sapiens serpin peptidase inhibitor, clade A (alpha-1 antiproteinase, antitrypsin), member 3 (SERPINA3), mRNA	Hs.534293
**CGB2**	−11.5101107	chr19	NM_033378	Homo sapiens chorionic gonadotropin, beta polypeptide 2 (CGB2), mRNA	Hs.567650
**KCNJ15**	−11.4040195	chr21	NM_170736	Homo sapiens potassium inwardly-rectifying channel, subfamily J, member 15 (KCNJ15), transcript variant 1, mRNA	Hs.411299
**PCDH1**	−11.2298908	chr5	NM_002587	Homo sapiens protocadherin 1 (PCDH1), transcript variant 1, mRNA	Hs.79769
**KRT19**	−10.4408865	chr17	NM_002276	Homo sapiens keratin 19 (KRT19), mRNA	Hs.654568
**PRSS2**	−10.3361156	chr7	NM_002770	Homo sapiens protease, serine, 2 (trypsin 2) (PRSS2), mRNA	Hs.728780
**LOC284837**	−10.0173389	chr21	NR_026961	Homo sapiens uncharacterized LOC284837 (LOC284837), non-coding RNA	Hs.592159

### Quantitative reverse transcriptase polymerase chain reaction (qRT-PCR) analysis

**Figure 4:**
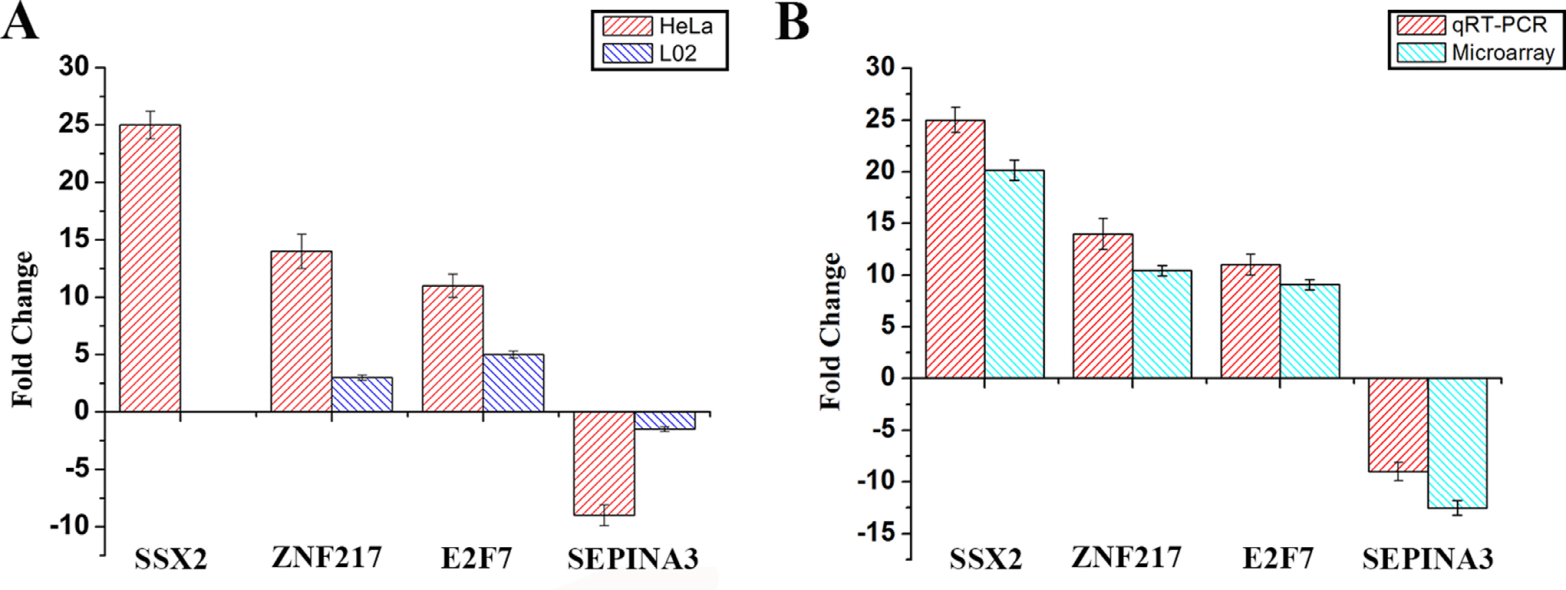
(**A**) The expression of four genes with qRT-PCR in Hela cells and L02 cells. (**B**) The expression of four genes in qRT-PCR study compared with microarray data. Housekeeping gene was beta-actin, which be used as an endogenous reference gene. The error bar represents standard deviation (SD) over three independent experiments.

qRT-PCR was performed to examine the expression levels of some important differentially expressed genes, including SSX2, ZNF217, E2F7 and SERPINA3. We have detected these four genes expression by RT-PCR incubated with Eu(NO_3_)_3_ solution in Hela and L02 cells, meanwhile DMEM instead of the Eu(NO_3_)_3_ solution in Hela and L02 cells as control with the same protocol. The results of the relative expression about four genes in reverse transcription (RT-PCR) were shown in Supplementary Figure 1. Figure 4 showed the results of the expression of four genes in qRT-PCR. It is indicated that ZNF217, E2F7 and SERPINA3 genes were expressed in L02 cells, the expression of ZNF217 and E2F7 were up-regulated, SERPINA3 genes was down-regulated (Figure 4A). Compared with Hela cells, the expressions of ZNF217, E2F7 and SERPINA3 genes of L02 cells was lower (Figure 4A). It is noted that SSX2 genes was not expressed in L02 cells, on matter incubated with Eu(NO_3_)_3_ solution or not (Supplementary Figure 1A).

Figure 4B showed the fold changes of expression levels of SSX2, ZNF217, E2F7 and SERPINA3 in qRT-PCR and microarray experiments. These three genes SSX2, ZNF217, E2F7 showed up-regulation in both qRT-PCR and microarray experiments. SSX2 gene showed down-regulation in both qRT-PCR and microarray experiments. It is obvious that the expression of four genes in qRT-PCR was consistent with the results from microarray study, indicating the reliability and reproducibility of the microarray in this study.

### GO function analysis of differential expressed genes

The GO as a dynamic controlled vocabulary can be used to describe the role of gene and protein in any organism with three categories information, such as cellular component, molecular function, and biological process.

**Figure 5:**
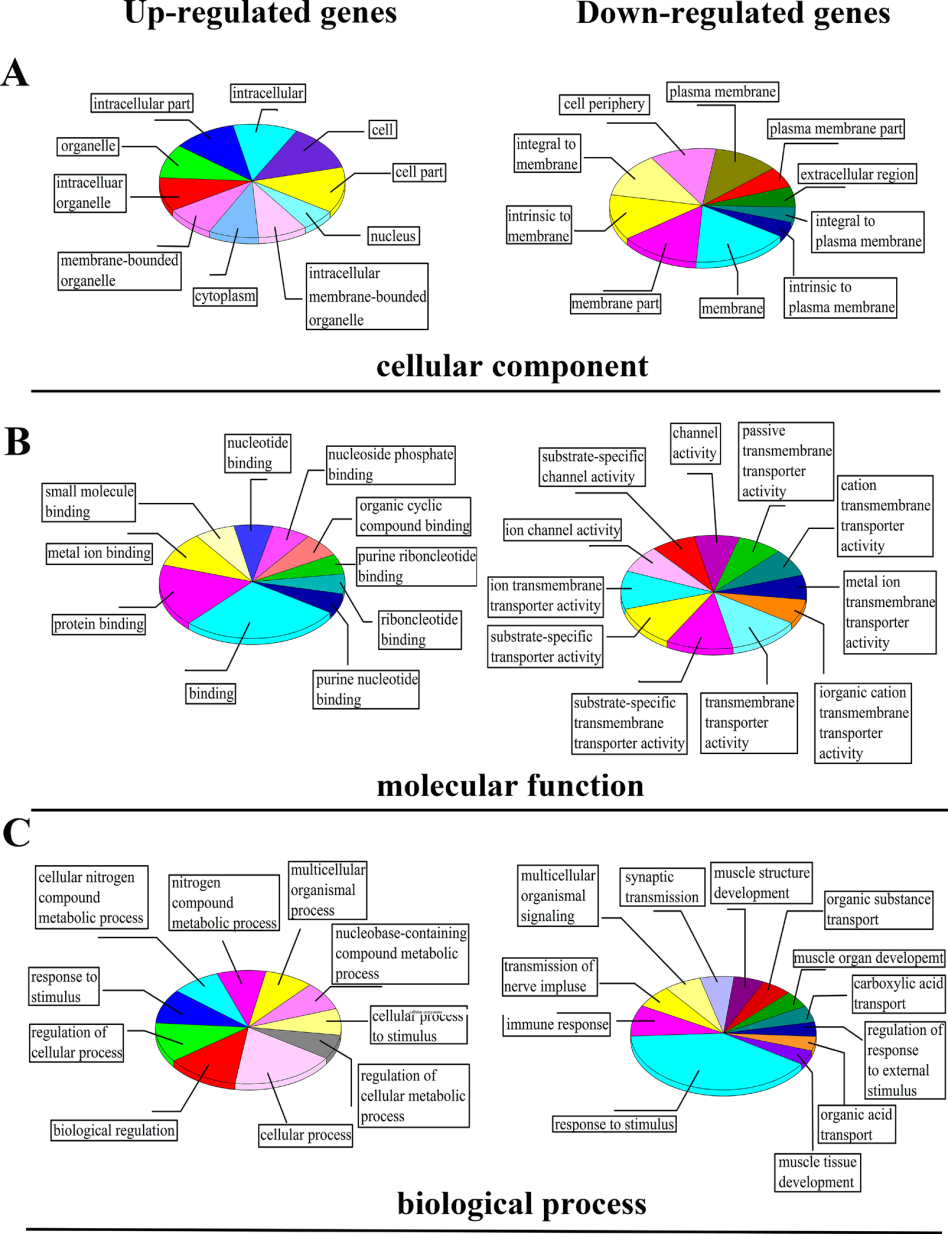
The classification results of differential expression of genes based on GO analysis: (**A**) cellular component, (**B**) molecular function and (**C**) biological process.

Among the three categories of GO, cellular component refers to that in the cell where a gene product is active [19]. In this study, we found that most genes expression happened in cell cytoplasm and membrane of organelle, with a little in the cell nucleus, especially with down-regulation genes (Figure 5A). Molecular function is defined as the biochemical activity of a gene product containing specific binding to ligands or structures [19]. As shown in Figure 5B, the differential expression of genes was mainly involved in cell binding and transmembrane transporter activity. It means the progress of biosynthesis Eu complex may be accomplished through changing the permeability of membrane and protein binding activity. Biological process refers to a biological objective to which the gene or gene product contributes, and this process may be accomplished via one or more ordered assemblies of molecular functions, which often involve a chemical or physical transformation [19]. In this study the genes were analyzed in relevant biological process by gene annotations. Figure 5C indicated that there are four main processes, including cellular metabolic process, cell response to stimulus, transport process and regulation of cellular process based on the biological process pertaining to ontology classification in up-regulated and down-regulated expression genes with the biosynthesis Eu complex.

**Figure 6:**
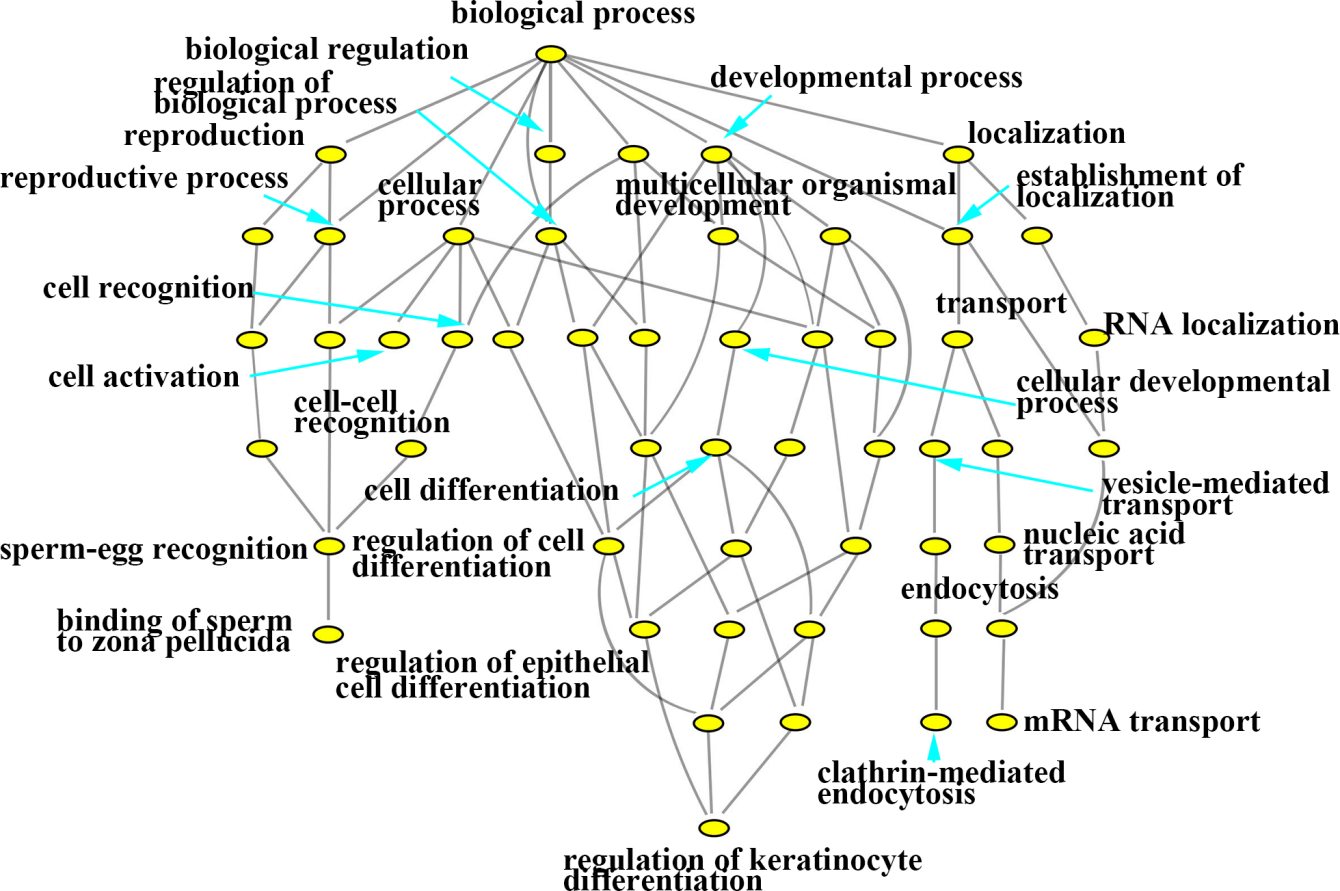
Hierarchical cluster tree built with up-regulated expression genes about biological processes

**Figure 7:**
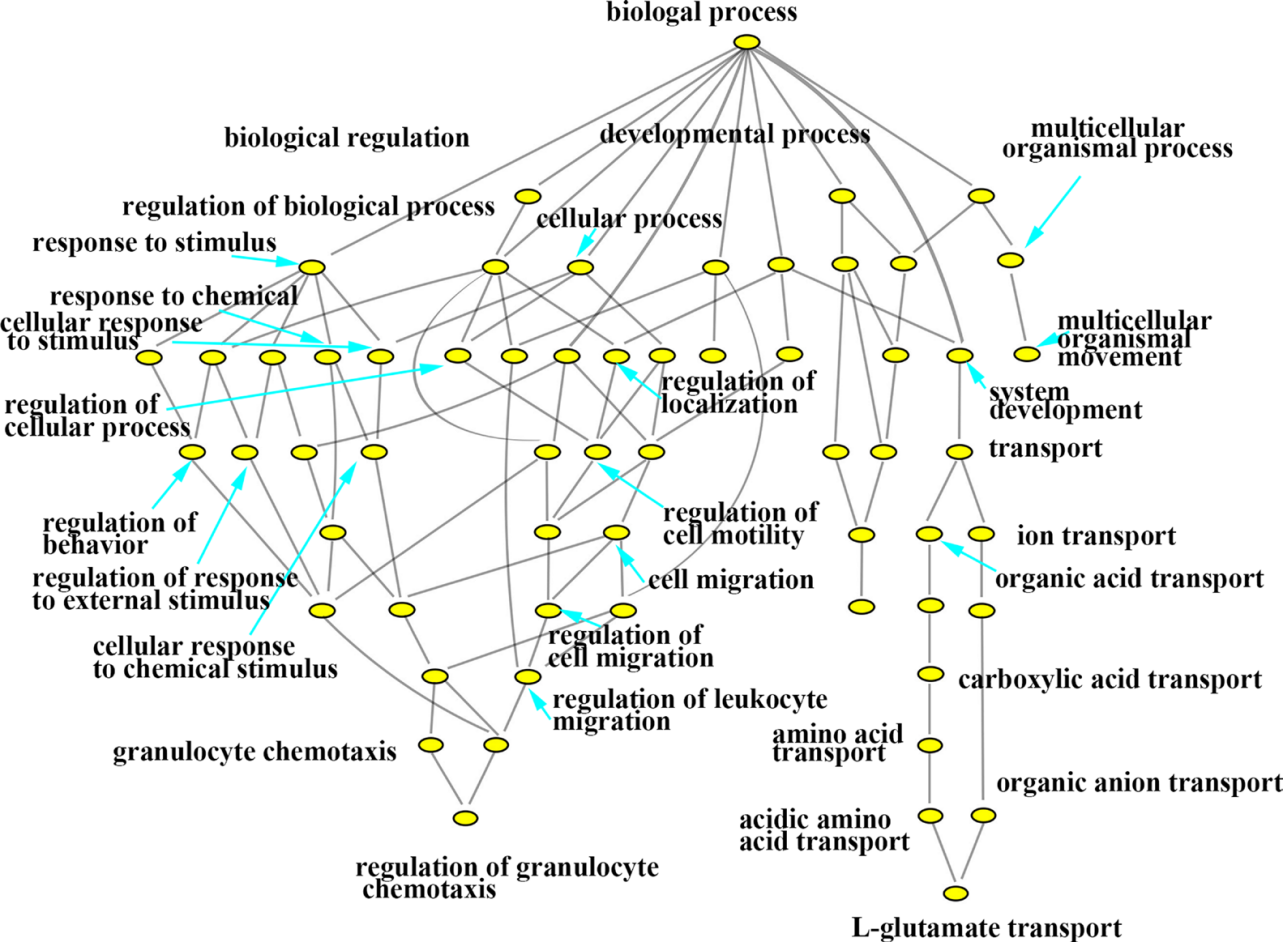
Hierarchical cluster tree built with down-regulated expression genes about biological processes

In this study, with the biosynthesis process of Eu complex, up-regulated genes and down-regulated genes (the date not show) were pertaining to individual biological process which could also be represented with a hierarchical tree structure (shown in Figure 6 and Figure 7). It is evident that some processes associated with self-biosynthesis were most significantly up-regulated (Figure 6), such as biological process for the regulation of cellular metabolic, response to oxygen levels and others. The genes ZNF217 and E2F7 were found to be involved in the regulation of cellular metabolic process. Meanwhile, as shown in Figure 7, it can be observed that some processes were significantly down-regulated in the self-biosynthesis process of fluorescence Eu complex, including those for cell response to stimulus, transportation process and so on. Besides, these processes related genes were also down-regulated, including SERPINA3 gene which has been discussed above.

### Biological pathways analysis

**Figure 8:**
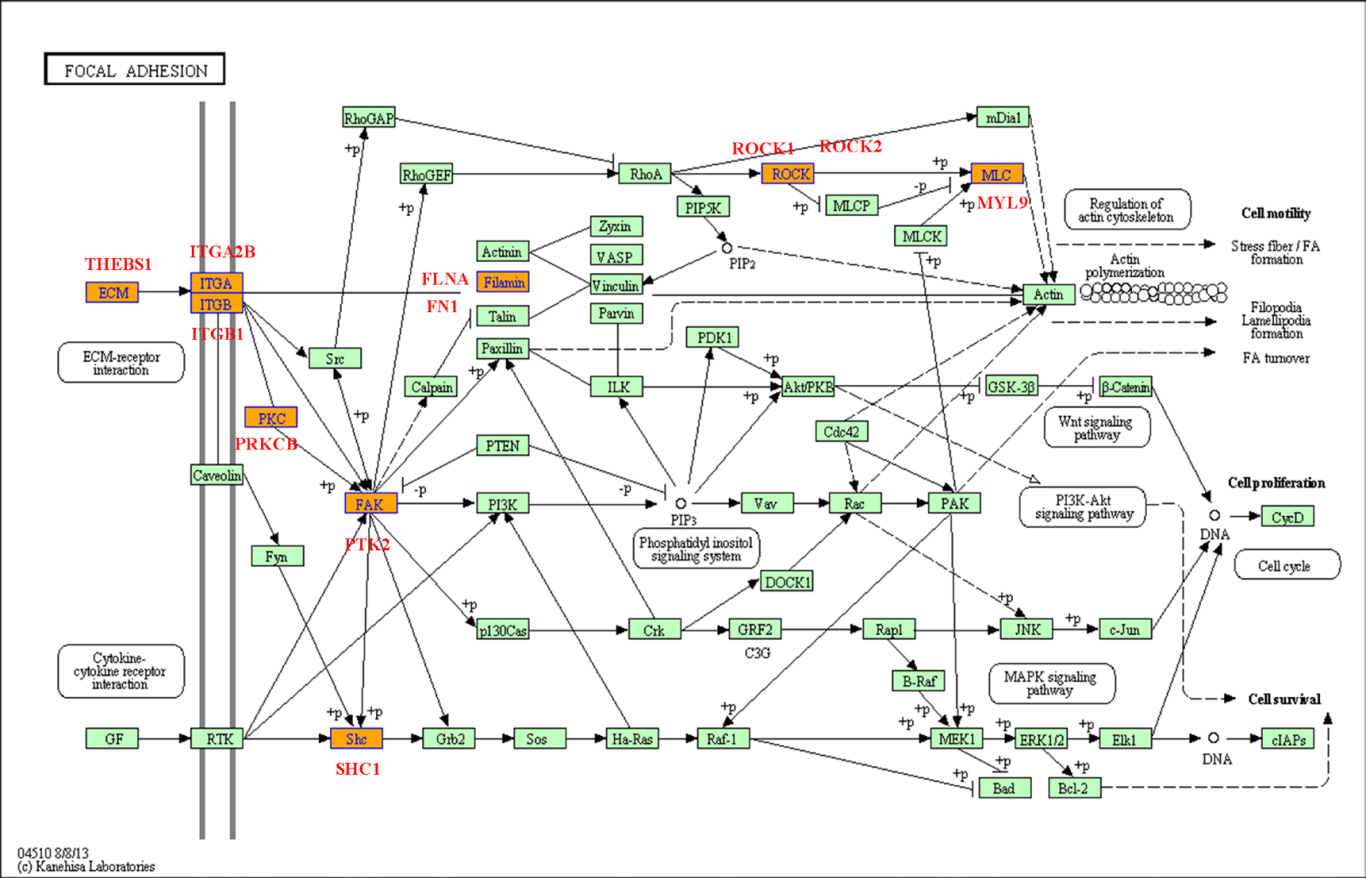
Focal adhesion pathway base on the latest KEGG database

**Figure 9:**
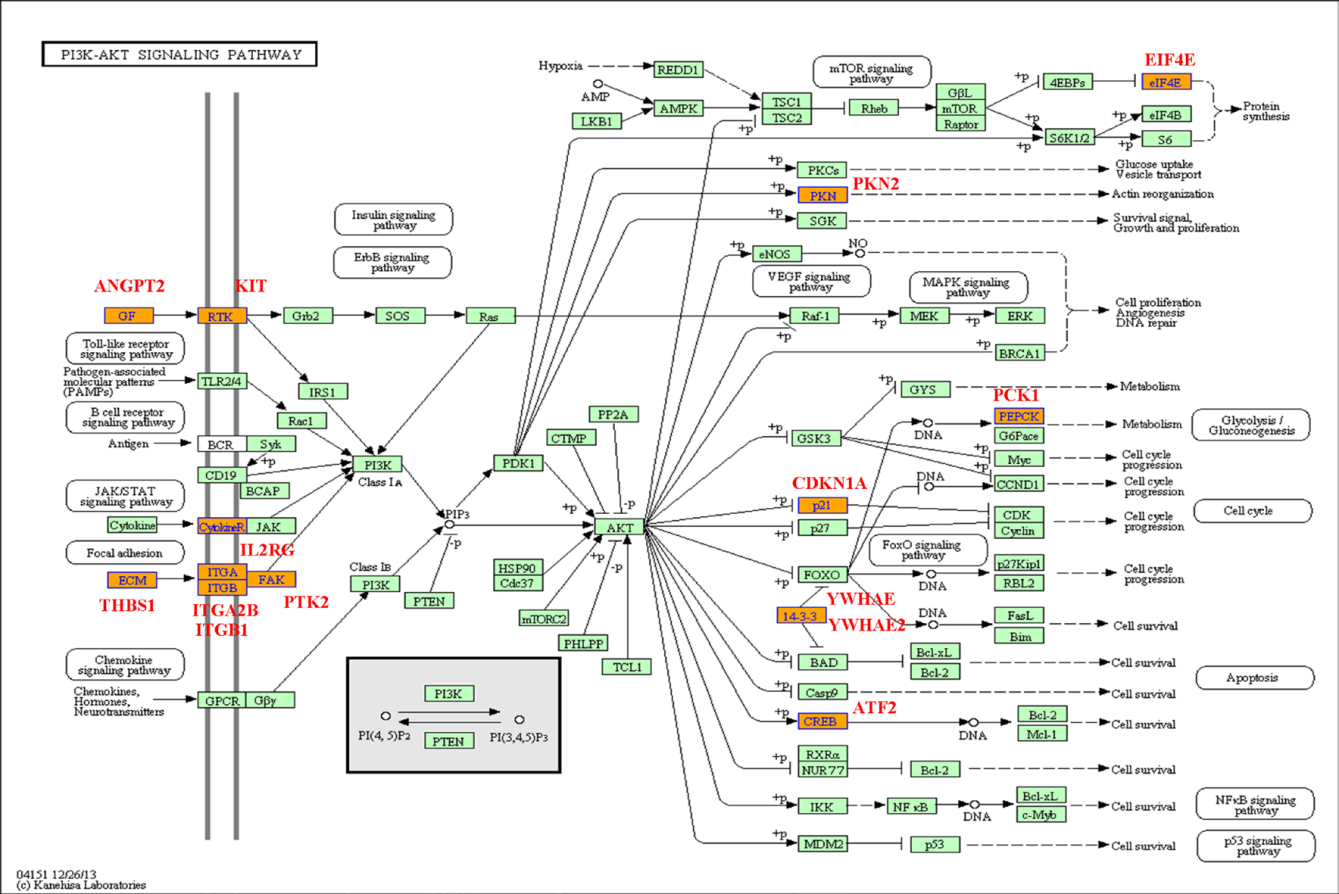
PI3K (phosphatidylinositol 3′ -kinase) -Akt signaling pathway base on the latest KEGG database

In general, the differentially expressed genes in a series of biological pathways are important for the kinds of physiological activities of cells. Based on the GO analysis, we have also analyzed the biological pathways with differential expressed genes related to the Eu complex biosynthesis, including the relevant biological pathways with up-regulation gens and other pathways with down-regulation genes (the data not shown). Among them, we will give some examples for the two important biological pathways including focal adhesion (as shown in Figure 8) and PI3K-Akt signaling pathway (as shown in Figure 9). Focal adhesions as specialized linking structures of cell and extracellular matrix (ECM) play significant roles in many important biological processes such as cell motility, cell proliferation and regulation of genes expression. The constituents of ECM are various signaling molecules. In the present study, there are 11 differentially expressed genes involved in the focal adhesion pathway.

PI3K-Akt signaling pathway regulates fundamental cellular functions such as transcription, translation, proliferation, growth, and survival, when it is activated by many kinds of cellular stimuli, through stimulating the PI3K isoforms. In our study, the PI3K-Akt signaling pathway involved 15 differentially expression genes like PKN2, PCK1, EIF4E and others.

## DISCUSSION

According to the confocal fluorescent imaging, fluorescent Eu complex could be spontaneously biosynthesized in cancerous cells by the molecular synthesis process, without in normal cells. This was possible because cancer cells have special biological environments and high levels of ROS, which may induce the up-regulation of GSH. In the relevant biosynthesis process, Eu (III) ions were reduced, resulting in the formation of the fluorescent Eu complex through oxidized GSH. On the side, NADPH in cells could also be participated in the reducing equivalents in biosynthetic reactions. In order to maintain the special biological microenvironments and physiological function, cancer cells could further up-regulate the expression of the related genes GSH and NADPH.

Several important function genes were found through GO analysis. The SSX2 gene expression was appeared significantly up-regulated in the process of self-biosynthesized fluorescence Eu complex in cancer cells. It is possible that the relevant biosynthesis process may affect the SSX2 genes expression. Previous research has identified that the SSX2 gene is one of the SSX gene family, which are expressed in many kinds of tumors or cancerous cells, but not in normal tissues or cells except for testis and very weak expression in the thyroid gland [20]. As our fluorescent study also indicated that the Eu complex could be spontaneously biosynthesized by cancerous cells, which cannot occur in non-cancerous cells (Figure 2). The mechanism of SSX genes activated in cancer cells is found to be related to the demethylation and lead to the reactivation of silenced genes and genomic instability.

SERPINA3 (Serpin peptidase inhibitor clade A member 3) is a member of the serpin super-family of protease inhibitors, and was significantly down-regulated in the process of biosynthesized fluorescence Eu complex. It is reported that SERPINA3 expression in cancer tissue is lower, where a high activity of relevant proteinases is linked to tumor growth, metastasis and aggressiveness [21–24].

ZNF217 located on chromosome 20q13.2 is frequently amplified in many tumors and cancer cells [25]. Previous studies have found that high levels expression of ZNF217 genes can attenuate apoptotic signals [26] and lead to activation of the phosphatidylinositol 3′ kinase (PI3K) pathway through increased levels of phosphorylated Akt [27]. AKT is a known modulator of the switch to glycolytic metabolism [28]. Akt activation by phosphorylation can contribute to improve the Glycolytic capacity, levels of ATP, and preventing release of cytochrome c from mitochondria. E2F7 gene as a new member of the human E2F family has an atypical structure feature distinguishing from other E2F subunits. Previous studies have shown that E2F7 as a transcriptional repressor can direct p53 target genes [29]. With the up-regulated E2F7 gene, p53-dependent effects on cells can be reinforced, which can promote pentose phosphate path, and generate much more NADPH for the biosynthesis of fluorescent Eu complex [30]. In fact, it is evident that the synthetic process, which the molecular precursor (Eu ion) was be transformed fluorescent Eu complex in the cancerous cells, may depend on some special biochemical characteristics and peculiar microenvironment in cancer cells, involving in the significance of NADPH-oxidases and GSH as well as metabolic pathway. With up-regulation of those genes could effectively provide beneficial environment for relevant biosynthetic process to produce fluorescence Eu complex, which could offer sufficient energy (ATP) and reductive substances (i.e. GSH and NADPH). More importantly, the results of genes differentially expressed are consistent with our previous studies, indicating that cancer cells treated with Eu ions could create necessary conditions for the biosynthesized fluorescent Eu complex.

To further investigated the mechanism of the biosynthesis in cancer cells, we have analyzed the biological pathways, and found two novel function pathways in the process. Focal adhesion pathway is important about adhesion between cells and ECM, which depend on the multi-molecular complex of junctional plaque proteins to transport the extracellular substance and initiate downstream signaling events. In the 11 differentially expressed genes involved in the focal adhesion pathway, some genes may play the vital functions. The up-regulation of THBS1 accompanying with the Eu complex biosynthesis can lead to the enhancement of the controlled cells and cell-matrix interactions by binding to ECM proteins and cell surface receptors [31]. The ITGA2B genes closely localize on chromosome 17 and code for αIIbβ3 [32], which participated in the Ca^2+^-dependent complex formation. Interestingly, it was reported that Eu ion could enter into the cells through the calcium ion channel [33]. Thus, the up-regulation of the ITGA2B in this study is consistent with this report. PTK2, also named FAK (Focal adhesion kinase), have important cellular functions, such as responding to signals from the extracellular matrix (ECM), regulating the cellular processes of proliferation and cell migration primarily through regulation of the cytoskeleton [34]. PTK2 was up-regulated in the process of self-biosynthesis fluorescence Eu complex, suggesting that it may enhance cell migration in the biosynthetic process.

In PI3K-Akt signaling pathway, PI3K can catalyze the production of phosphatidylinositol-3, 4, 5-triphosphate (PIP3) at the cell membrane and in turn help to activate Akt. As above discussion, Akt activated can control key cellular processes by phosphorylating substrates such as protein synthesis, metabolism, and cell cycle. In this study, PKN2 genes have the special function in 15 differentially expressed genes. Previous reports indicated that PKN2 was relatively highly expressed and had a significant role in the migration and invasion of cancer cells [35]. In this study, PKN2 was up-regulated with the Eu complex informed, suggesting that this gene can lead to the migration in Hela cell, which is also benefit to increase completed the relevant biosynthesis process.

**Figure 10:**
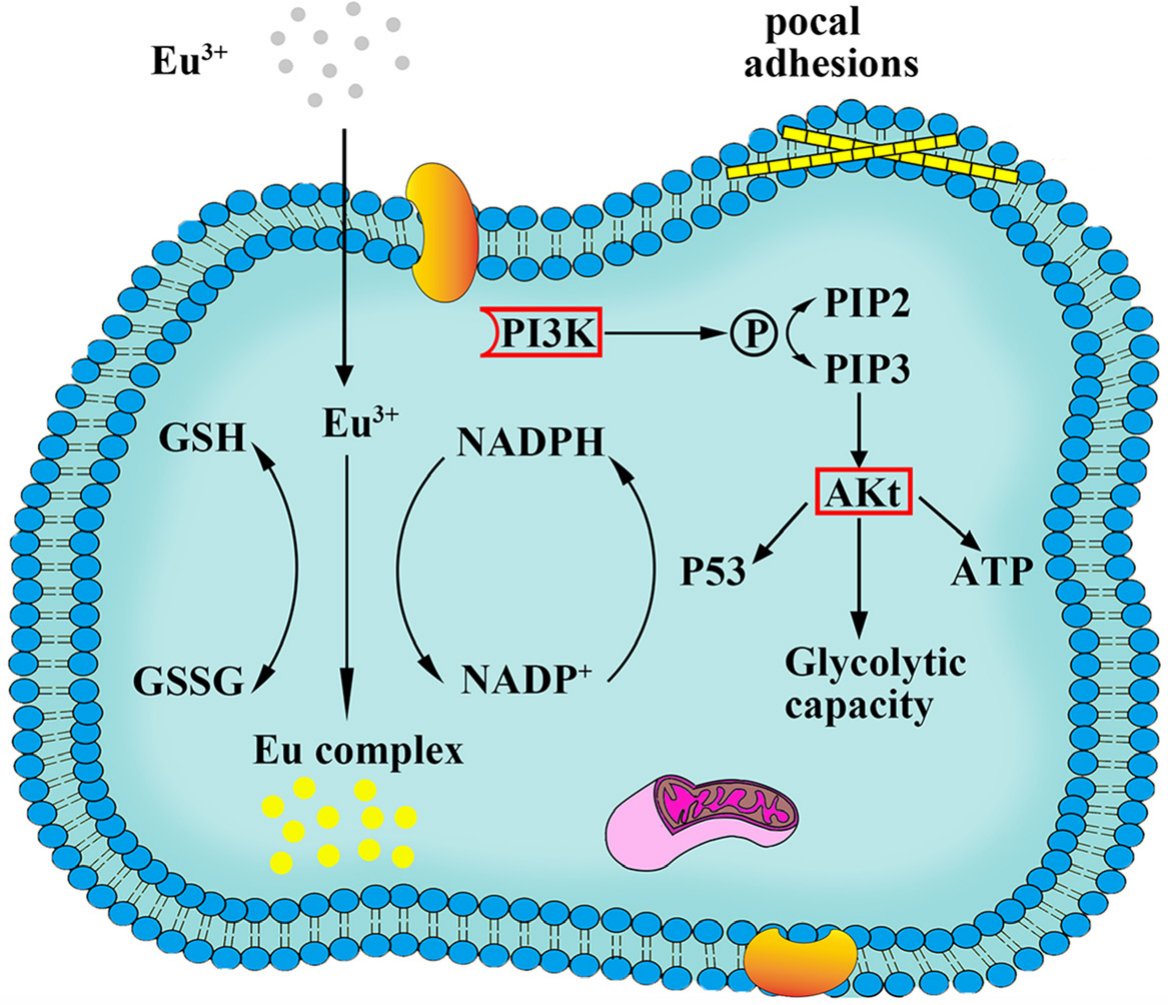
Scheme of the process of biosynthesized Eu complex

As described above, the possible molecular mechanism of the biosynthesis fluorescence Eu complex in cancer cells could be explored through combining the gene microarray technology with bioinformatics analysis. As shown in Figure 10, it is evident that Eu ion incubated with Hela cells stimulated the receptor in the cell membrane, inducing the up-regulation of ITGA2B to enter the cell membrane by utilizing calcium ion channel, then in the cytoplasm, the process of molecular precursor (Eu ion) to fluorescent Eu complex in cancer cells could readily occur, where a lot of genes were up-regulation which involved in cell recognition, cell proliferation, cell migration and relevant biosynthetic process. The up-regulated expression of ZNF217 and E2F7genes indicated that the synthetic process is likely to be connected with glycolytic metabolism and NAD(P)H-oxidases metabolic pathway, activated Akt by phosphorylation to improve the glycolytic metabolism and directly target p53 genes to increase p53-dependent effects. The activation of these physiological reaction would help cells to obtain enough energy, reducing agents and NAD(P)H for the *in situ* biosynthesis of fluorescent Eu complex. In addition, focal adhesion function may increase cell adhesion, proliferation and migration. More importantly, the significance of the up-regulation of SSX2 genes suggested fluorescent Eu complex could only be biosynthesized in cancer cells, without in normal cells.

## MATERIALS AND METHODS

### Cell culture and MTT assays

Cervical carcinoma (Hela) cells were purchased from Shanghai Institute of Cells, Chinese Academy of Sciences. Hela cells were cultured in DMEM (HyClone, USA) supplemented with 10% fetal calf serum (HyClone, USA) and 1% penicillin-streptomycin (Hyclone, USA) in 37°C incubator with 5% CO_2_.

The cytotoxicity on Hela cells was measured by using methyl thiazolyl tetrazolium(MTT) assays. MTT test is performed according to the literature [18].

### Confocal fluorescence imaging of cells

0.01 mmol/L Eu(NO_3_)_3_ solution was incubated with Hela cells for 24 h, and the fluorescence microscopy images were obtained through laser confocal fluorescence microscope. Under the continuous wave laser at 488 nm as excitation, while fluorescence emission at 500–650 nm was collected as output signal.

### Total RNA isolation

After the treatment with Eu(NO_3_)_3_ solution (0.01 mmol/L) for 24 h, the total RNA from the sample was extracted from Hela cells by using a TRIzol^®^Reagent total RNA isolation Kit (Invitrogen life technologies, USA) according to the instructions provided by the manufacturer. Total RNA concentration and purity were quantified using the NanoDrop ND-1000 by the absorbance ratio 260:280 nm, and the RNA integrity was assessed by appearance of distinct 28S and 18S bands of ribosomal RNA using standard denaturing agarose gel electrophoresis.

### Gene expression study

Total RNA was amplified and then transcribed into fluorescent cRNA according the manufacturer's Agilent's Quick Amp Labeling protocol (version 5.7, Agilent Technologies). Cy3 was used for cRNA labeling. The labeled cRNAs were hybridized onto the Whole Human Genome Oligo Microarray (4 × 44 K, Agilent Technologies). After hybridizing the samples for 17 h at 65°C, and then washed the slides. The arrays were scanned by the Agilent Scanner G2505C. The acquired array images were analyzed through utilization the Agilent Feature Extraction software (version 11.0.1.1). Quantile normalization and subsequent data processing were performed by using the GeneSpring GX v11.5.1 software package (Agilent Technologies).

### GO and pathway analysis of gene expression data

Gene Ontology (GO) analysis is a special functional analysis associating differentially expressed genes with GO categories. The GO categories are derived from Gene Ontology (www.geneontology.org), which comprise three structured parts (molecular function, biological processes, and cellular component) of defined terms that describe gene product attributes. Based on the KEGG (Kyoto Encyclopedia of Genes and Genomes) database, pathway analysis was performed through differential expression genes. It allows us to understand and determine the significant biological pathways which have a significant enrichment of differential expression genes. The differentially expressed genes were identified by fold-change filtering. In general, fold change ≥ 2 or fold change ≤ −2 was considered as significantly differential expression. Therefore, GO analysis and pathway analysis were performed to effectively reveal the biological functions of differential expression genes.

### qRT-PCR experiments

On account of bioinformatics analyses, four important genes (SSX2, ZNF217, E2F7, SERPINA3) were selected for qRT-PCR experiments. Total RNA isolation from Hela and L02 cells and cDNA synthesis. Eu(NO_3_)_3_ solution was incubated with Hela and L02 cells for 24h. TRIzol reagent was used to isolate total RNA from the Hela and L02 cells, according to the instructions provided by the manufacturer. Then total RNA was converted to complementary DNA (cDNA) by using PrimeScript RT Master Mix kit (Takara, Dalian, China), according to the manufacturer's protocol, in order to subsequent PCR amplification.

qRT-PCR experiment was measured on a 7500 Real-Time PCR system (Applied Biosystems Inc., USA) with SYBR Premix Ex Taq^TM^II (Takara, Dalian, China). For each gene, qRT-PCR reactions were carried out with three biological replicates. As an internal control, we used β-Actin as housekeeping reference gene. Gene relative expression ratios were calculated using the log_2_2^−ΔΔCt^method. The final quantification cycle values were the means of three replicates.

All of the primers in this study were listed in Supplementary Table 1. All the primers characterized by lengths of 19–24 bp, melting temperatures of 54–61°C, and GC contents of 43%–59%.

## CONCLUSIONS

In summary, the present study could provide a relatively integrated overview of the biosynthesis fluorescence Eu complex in Hela cells, by the whole-genome expression profiles with gene microarray. GO analysis showed that the fluorescence Eu complex self-biosynthesis in Hela cells involved in large rang of functional genes which belong to cellular component, molecular function and cellular biological process. The biosynthesized Eu complex process changes the expression level of genes such as SSX2, ZNF217and E2F7, which are pertaining to obtaining enough energy, reducing agents and NAD(P)H through interfering cell proliferation, glycolytic metabolism and cellular biosynthetic. Furthermore, the possible molecular mechanisms of the self-biosynthesis of fluorescence Eu complex have been explored by combining GO and pathway analysis. Considering the whole-genome expression profiles analysis, it is evident that Eu complex formation could change glycolytic metabolism and NAD(P)H-oxidases metabolic pathway, increasing cell proliferation, activation signal transport in cancer cells. The integrative analysis of the expression profiles may help to identify relevant synthesis biological process, which could further beneficial for the cancer diagnostics and treatment.

## SUPPLEMENTARY TABLE AND FIGURE


