# Supplementary material for: Genome-wide functional analysis on the molecular mechanism of specifically biosynthesized fluorescence Eu complex
Source: Oncotarget. 2017 Jul 1;8(42):72082–95. doi: 10.18632/oncotarget.18914 (PMC5641113; doi:10.18632/oncotarget.18914)
Supplement: Supplementary file 1 [file oncotarget-08-72082-s001.pdf]

Genome-wide functional analysis on the molecular mechanism of specifically biosynthesized fluorescence Eu complex

Supplementary Materials

Supplementary Table 1: Primer sequences used in qRT-PCR

qRT-PCR primer	Forward	Reverse
SSX2	GTGCTCAAATACCAGAGAAGATC	TTTTGGGTCCAGATCTCTCGTG
SERPINA3	TGCCAGCGCACTCTTCATC	TGTCGTTCAGGTTATAGTCCCTC
E2F7	GGAAAGGCAACAGCAAACTCT	TGGGAGAGCACCAAGAGTAGAAGA
ZNF217	GAGAAGCGAATGGTGAAAGC	CAGCGCTCAAGTATGCAAAA
β-Actin	CTCCTCCTGAGCGCAAGTACTC	CGGACTCGTCATACTCCTGCTT

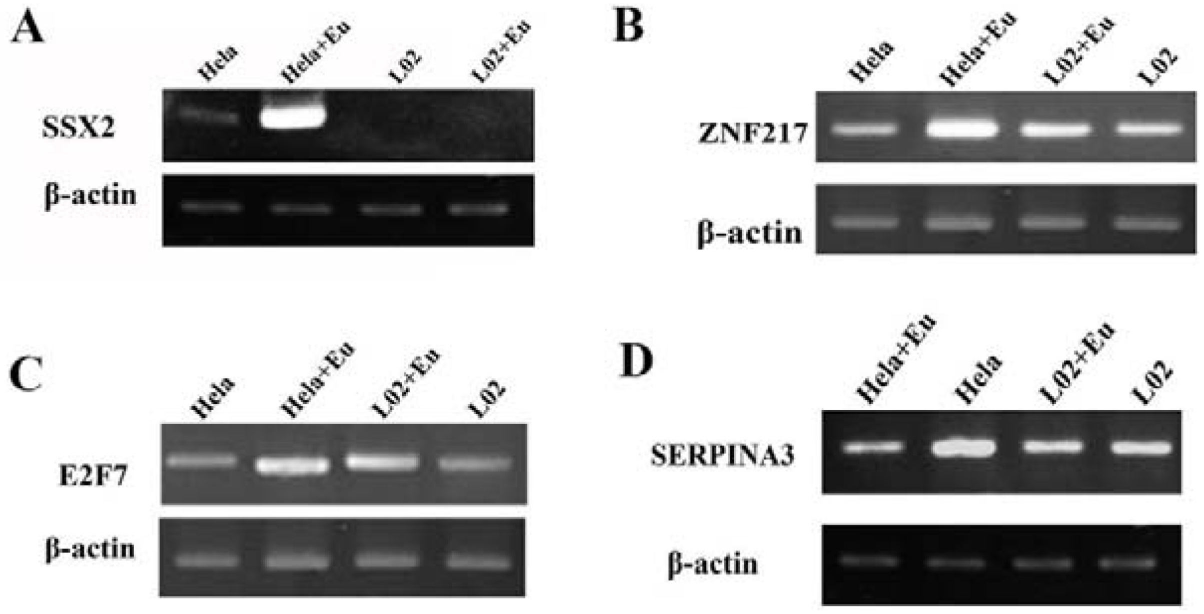

Supplementary Figure 1: The results of RT-PCR for the expression of four genes. (**A**) SSX2; (**B**) ZNF217; (**C**) E2F7; (**D**) SERPINA3. Housekeeping gene was beta-actin as an endogenous reference gene.
